# Supplementary material for: fNIRS-Based Differences in Cortical Activation during Tool Use, Pantomimed Actions, and Meaningless Actions between Children with and without Autism Spectrum Disorder (ASD)
Source: Brain Sci. 2023 May 29;13(6):876. doi: 10.3390/brainsci13060876 (PMC10296505; doi:10.3390/brainsci13060876)
Supplement: Supplementary file 1 [file brainsci-13-00876-s001.zip › brainsci-2366445-supplementary.pdf]

fNIRS-based Differences in Cortical Activation During Tool-use, Pantomimed Actions, and Meaningless Actions between Children with and without Autism Spectrum Disorder (ASD)

Wan-Chun Su,[1,2] McKenzie Culotta,[1,2] Jessica Mueller,[3] Daisuke Tsuzuki,[4] Anjana N. Bhat [1,2,5*]

Supplementary Table S1. Spatial registration and assignment for right and left hemisphere channels.

Side	Ch	MNI's coordinate system			MIFG regions			IPL regions			MSTG regions		Assigned region
		X	Y	Z	Inferior frontal gyrus	Middle frontal gyrus	Precentral gyrus	Postcentral gyrus	Supramarginal gyrus	Angular gyrus	Middle temporal gyrus	Superior temporal gyrus	
left	1	-51.3	0.3	53.7		18.0	81.6	0.4					MIFG
	2	-56.7	-29.7	54.3				30.0	70.0				IPL
	3	-53.0	17.7	39.7	0.4	81.4	18.3						MIFG
	4	-62.3	-14.7	43.7			4.2	71.0	24.7				IPL
	5	-63.0	-43.3	45.3					70.6	29.4			IPL
	6	-63.7	2.7	29.3			69.2	30.8					MIFG
	7	-68.0	-28.7	32.7					98.7			1.3	IPL
	8	-60.0	20.3	14.3	89.6		10.4						MIFG
	9	-69.0	-13.3	18.3				70.7	9.8			19.6	Excluded*
	10	-68.7	-43.7	19.3					32.8	0.6	1.3	65.3	MSTG
	11	-63.0	0.7	0.3	1.3		21.2	3.6			2.0	71.9	MSTG
	12	-71.0	-29.7	2.7							56.0	44.0	MSTG
right	13	54.7	-32.7	58.3				8.3	83.0	8.7			IPL
	14	52.3	-1.7	55.7		1.7	60.8	37.5					SA
	15	60.7	-48.7	48.3					15.6	84.4			IPL
	16	64.0	-16.3	45.7				15.9	54.1				IPL
	17	54.7	15.3	40.7	2.9	48.9	48.2						MIFG
	18	70.0	-32.3	34.3					88.2	11.8			IPL
	19	66.3	0.3	31.3			63.2	35.8	1.0				MIFG
	20	68.0	-49.3	20.3					3.9	33.1	26.9	36.1	MSTG
	21	70.0	-17.3	18.3				33.2	20.8		46.0		Excluded*
	22	63.3	15.7	14.3	12.3		87.7						MIFG
	23	73.0	-35.3	3.3							66.0	34.0	MSTG
	24	68.3	-3.3	-0.3				4.3			11.6	84.1	MSTG

Supplementary Table S2. The means and SEs of HbO_2 concentration in children with and without ASD during Hammer, Pantomime, and Meaningless conditions.

**Group activation data**	**Hammer**		**Pantomime**		**Meaningless**	
	**Mean**	**SE**	**Mean**	**SE**	**Mean**	**SE**
**TD**						
Left hemisphere						
MIFG	0.046	0.005	0.052	0.006	0.048	0.005
MSTG	0.062	0.007	0.058	0.006	0.068	0.007
IPL	0.020	0.005	0.029	0.006	0.020	0.005
Right hemisphere						
MIFG	0.007	0.005	0.027	0.006	0.017	0.005
MSTG	-0.002	0.006	0.017	0.006	0.016	0.006
IPL	-0.022	0.005	-0.002	0.006	-0.010	0.005
**ASD**						
Left hemisphere						
MIFG	0.042	0.006	0.063	0.007	0.044	0.006
MSTG	0.034	0.007	0.038	0.007	0.023	0.007
IPL	0.025	0.005	0.027	0.005	0.021	0.005
Right hemisphere						
MIFG	0.039	0.005	0.043	0.006	0.030	0.006
MSTG	0.010	0.007	0.037	0.007	0.010	0.006
IPL	0.007	0.007	0.025	0.006	0.015	0.007

Supplementary Table S3. The post-hoc analyses for the Group x Condition x Hemisphere x Region 4-way interaction.

**Comparison**	**Significant *p* values**	**Direction of effect**
**Group related difference**		
Hammer condition		
Left MSTG	0.006	TD > ASD [a]
Right MIFG	0.000	ASD >TD [a]
Right IPL	0.001	ASD > TD [a]
Pantomime condition		
Left MSTG	0.031	TD > ASD [b]
Right MSTG	0.029	ASD > TD [b]
Right IPL	0.002	ASD > TD [a]
Body on Object condition		
Left MSTG	<0.001	TD > ASD [a]
Right IPL	0.004	ASD > TD [a]
**Condition related difference**		
TD group		
Right MIFG	<0.001	P > H [a]
	0.029	P > B [b]
Right MSTG	0.001	P > H [a]
	0.003	B > H [a]
Right IPL	0.001	P > H [a]
	0.037	B > H [b]
ASD group		
Left MIFG	0.016	P > H [b]
Right MIFG	0.017	P > B [b]
Right MSTG	0.003	P > H [a]
	0.001	P > B [a]
Right IPL	0.047	P > H [b]
**Hemisphere related difference**		
TD group		
Hammer, MIFG	<0.001	L > R [a]
Hammer, MSTG	<0.001	L > R [a]
Hammer, IPL	<0.001	L > R [a]
Pantomime, MIFG	<0.001	L > R [a]
Pantomime, MSTG	<0.001	L > R [a]
Pantomime, IPL	<0.001	L > R [a]
Body on Object, MIFG	<0.001	L > R [a]
Body on Object, MSTG	<0.001	L > R [a]
Body on Object, IPL	<0.001	L > R [a]
ASD group		
Hammer, MSTG	0.002	L > R [a]
Hammer, IPL	0.008	L > R [a]
Pantomime, MIFG	0.003	L > R [a]
Body on Object, MIFG	0.015	L > R [a]

a. The *p* values survived the FDR correction
b. The *p* values < 0.05, but not survived the FDR correction

Supplementary Table S4. Correlations between cortical activation and VABS scores.

**r-values**	**VABS-Communication**			**VABS-Daily Living**			**VABS-Socialization**			**VABS-Total**		
	**H**	**P**	**M**	**H**	**P**	**M**	**H**	**P**	**M**	**H**	**P**	**M**
**TD Children without ASD**												
Left hemisphere												
MIFG	-0.200*	-0.029	0.095	-0.014	0.103	0.096	0.029	0.063	-0.066	0.007	0.123	0.083
MSTG	*0.170*	-0.209*	0.132	-0.013	-0.063	-0.187*	*-0.185*	0.084	-0.237*	-0.030	-0.013	-0.180
IPL	0.120	**0.266****	0.097	0.113	**0.250****	0.144	*-0.172*	-0.140	-0.111	0.018	0.146	0.069
Right hemisphere												
MIFG	0.059	0.096	0.240*	0.120	0.130	**0.266****	**0.493****	**0.283****	**0.392****	**0.333****	**0.254****	**0.420****
MSTG	0.043	0.145	0.104	-0.039	-0.001	-0.179	**0.244****	0.138	*0.166*	0.129	0.115	0.035
IPL	**-0.251****	-0.001	0.124	-0.215*	-0.057	0.037	**0.277****	*0.161*	0.209*	-0.029	0.065	0.151
**Children with ASD**												
Left Hemisphere												
MIFG	0.133	-0.054	0.162	0.079	-0.147	-0.022	-0.058	-0.108	-0.121	0.049	0.125	-0.003
MSTG	0.253*	*-0.195*	-0.173	*0.210*	-0.104	-0.041	0.177	-0.220*	-0.096	0.229*	-0.169	-0.095
IPL	-0.135	-0.219*	-0.125	*-0.210*	-0.170	-0.108	**-0.341****	0.011	-0.045	-0.245*	-0.133	-0.090
Right Hemisphere												
MIFG	0.257*	-0.111	0.072	0.171	-0.141	-0.036	0.047	-0.267*	-0.247*	0.170	-0.184	-0.075
MSTG	**0.337****	-0.012	0.104	*0.196*	-0.072	0.089	-0.001	-0.263*	-0.190	0.180	-0.127	0.007
IPL	-0.059	0.247*	0.017	0.021	*-0.185*	0.022	-0.016	*-0.185*	0.037	-0.011	-0.213*	0.040

r values are presented in this figure. *indicates $p < 0.05$; ** indicates $p < 0.01$. Bolded font with shading indicates p values that survived FDR corrections. Italic font indicates p values between 0.05 and 0.1. H = hammer condition; P = Pantomime condition; M = Meaningless condition. Blue highlighting shows the ROIs associated with tool use for children with and without ASD given the multiple correlations observed between VABS scores and activation data.

Abbreviations

ASD, autism spectrum disorder; IPL, inferior parietal lobe; STS, superior temporal sulcus; MTG, middle temporal gyrus; IFG, inferior frontal gyrus; fMRI, functional Magnetic Resonance Imaging; TD, typically developing; EEG, electroencephalogram; ERD, event related desynchronization; fNIRS, functional near infra-red spectroscopy; SIPT-PP, Sensory Integration & Praxis Test, postural praxis; SIPT-BMC, Sensory Integration & Praxis Test, bimanual coordination subtests; IQ, Intelligence Quotient; BOT-2, Bruininks-Oseretsky Test of Motor Proficiency; HbO_2, Oxygenated Hemoglobin; SE, Standard Error; MSTG, Middle and Superior Temporal gyri; MIFG, Middle and Inferior Frontal Gyri; ADOS, Autism Diagnostic Observation Schedule, Social Affect; ADOS-RRB, Autism Diagnostic Observation Schedule, Repetitive Behaviors; ROI, Region of Interest; SES-Child = Hollingshead Four-Factor Index of Socioeconomic Status; SCQ = Social Communication Questionnaire; VABS-II = Vineland Adaptive Behavior Scale - 2nd Edition; M = Male, F = Female; C = Caucasian, A = Asian, BC= Black-Caucasian, AC = Asian-Caucasian, AI = American Indian. R = right, L = left; MNI, Montreal Neurological Institute; LPBA, LONI Probabilistic Brain Atlas; FDR, False Discovery Rate.
